# Alcohol prices, the April effect, and the environment, in violence-related injury in England and Wales

**DOI:** 10.1007/s10198-023-01583-w

**Published:** 2023-03-29

**Authors:** Kent Matthews, Saeed Heravi, Peter Morgan, Nicholas Page, Jonathan Shepherd, Vaseekaran Sivarajasingam

**Affiliations:** 1 Cardiff Business School, Cardiff University, Colum Drive, Cardiff, CF10 3EU UK; 2 School of Dentistry, Cardiff University, University Hospital Wales, Heath Park, Cardiff, CF14 4XY UK; 3 School of Social Sciences, Cardiff University, Spark, Maindy Road, Cardiff, CF24 4HQ UK; 4 Nottingham University Business School China, University of Nottingham Ningbo China, 199 Taikang East Road, Ningbo, 315100 China

**Keywords:** Violent injury, Emergency departments, Price of alcohol, Tax policy, K40, I30, C50

## Abstract

Violent injury varies widely across England and Wales as does the price of alcohol. While the links between alcohol consumption and violence are well established in the medical and epidemiological literature, a causal link is questionable. This paper cuts through the causative argument by reporting a link between the general price of alcohol and violence-related injury across the economic regions of England and Wales. It examines the influence of the real price of alcohol and identifies an ‘April effect’ that coincides with the annual uprating of alcohol prices for excise duties, on violence-related injuries recorded at Emergency Department attendance. The data are monthly frequency of violent injury rates covering the period 2005–2014 across the economic regions. The principal finding is that a one-way relationship between the real price of alcohol and violent injury is established, and tax policy can be used to reduce the incidence of violent injury and the associated health costs.

**Supplementary Information:**

The online version contains supplementary material available at 10.1007/s10198-023-01583-w.

## Introduction

The link between alcohol and violence is heavily researched in epidemiology with an emphasis on the effects of heavy drinking or alcoholism and violent behaviour. The literature evidences strong correlations between alcohol consumption and violence.^1^ In the recent economics literature Luca et al. [34] report the link between excessive alcohol consumption and domestic violence.^2^ Yet, despite this connection being considered robust, there is no consensus on causation.^3^ The association can be viewed in three ways. First, alcohol misuse may cause violent behaviour. Second, people with a violent tendency may turn to alcohol as part of their antisocial behaviour. Or third, both alcoholism and violence share an unobserved common pathology.

Markowitz [36, 37] has pioneered investigations into a link between the price of alcohol and violence. This line of reasoning cuts through the causation debate by arguing that, since violence does not cause the price of alcohol, it follows that the relationship between the price of alcohol and violence must occur through alcohol consumption. Several studies have examined this relationship in the case of the USA using survey data,^4^ and, in the UK, two studies have examined this relationship using Emergency Department (ED) data.^5^

The official data on violent crime in England and Wales are the Crime Survey for England and Wales (CSEW) and Police Recorded Crime Statistics (RCS). While the CSEW provides a comprehensive snapshot of crime typology, its annual frequency means that statistical trends must be analysed from a long-term viewpoint. It is generally accepted that the CSEW and Recorded Crime Statistics, both provided by the Home Office, under-record certain types of violent crime—notably stranger and domestic violence.^6^

This paper utilises violent injury data from EDs of regional hospitals in England and Wales. Monthly violent injuries data from ED departments are obtained for the purposes of this study and represent the only objective data source of violence since they do not depend on the perception that a crime has been committed or on police reporting. The availability of relatively high frequency data affords the analysis of violence-related injuries as an alternative measure of violent crime that incorporates trend, seasonal, and other systematic factors.

The purpose of this paper is to demonstrate a causal link between alcohol consumption and violent injury using alcohol prices as an instrument. It goes further than Matthews et al. [42], since it extends the analysis to cover the general price of alcohol as well as prices of other alcohol types and goes further than Page et al. [46] by examining the effect of the April uprating of alcohol duty changes imposed in the UK following the Budget statement. Here, we develop a dynamic econometric model that explains violence-related injury victims in terms of the real price of alcoholic beverages with a strong ‘April effect’ on violent injury from the uprating of excise duties on alcohol in the March Budget. Additionally, environmental factors, such as recorded rainfall and temperature, account for exogenous factors.

The paper is organised in the following way. The next section reviews the literature on the link between alcohol consumption and violence. Sect. “An analytical framework” outlines the analytical framework. Sect. “Data” describes the data. Sect. “Econometric model” presents the main empirical results. Sect. “Robustness tests” presents robustness tests using hospital level data. Sect. “Discussion” concludes.

## The background

### Alcohol and violence

According to CSEW 2016, victims of violent crime judged the perpetrator of violence to be under the influence of alcohol in 40% of incidents. Alcohol was mostly associated with ‘stranger violence’ (62%)—reflecting the high incidence of violent injury in or near pubs and night clubs. Similarly, in the USA, 40% of criminal offenders reported using alcohol at the time of the offence [23]. The association between alcohol consumption and violence is well documented in the epidemiological literature.^7^

Links between alcohol consumption and injury by assault have been investigated by means of case–control studies, demonstrating a positive dose effect on seriousness of injury [53]. Shepherd et al. [54] and Shepherd and Brickley [52] discovered the link between injury and binge drinking (> 8 alcohol units). Links between alcohol dependence and injury have been found only in victims aged over 35 years [55]. The mechanism for the link between ‘binge’ drinking and injury include physical handicap, poor decision-making, isolation in vulnerable settings, and signals of immunity to prosecution [51].

There are several explanations why alcohol and violence are linked. One theory is that there is a psychopharmacological disinhibition process by which alcohol alters behaviour [48, 49]. By this explanation, a provocative or threatening event can interact with a disinhibition process arising from the psychopharmacological effects of alcohol. Some explanations centre on the biological makeup of people (mostly men), which causes them to behave violently after alcohol intake [32].

The ‘deviance disavowal’ theory suggests people use alcohol as an excuse for aberrant behaviour, loss of inhibition, and release of violent tendencies [14, 20, 21]. Other explanations centre on the planned use of pharmacological effects, where alcohol is consumed as a rational means of giving a person ‘Dutch courage’ [6, 48]. People are more likely to commit acts of violence when under the influence of alcohol than otherwise.

Finally, a common factor could exist that results in both drinking and violent behaviour arguing that risk factors and lifestyles encourage alcohol consumption and independently increase the risk of involvement in violent activity.^8^ Studies invoking such common factors include Ensor and Godfrey [12], White et al. [61], Fergusson et al. [17], and Fergusson and Horwood [16]. Although these studies suggest a possible causal association, there remains no consensus as to the causal link [59].

### Alcohol taxes and violence

Some evidence of a causal link can be gleaned from the economics literature. Using the National Family Violence Survey in the USA, Markowitz and Grossman [40, 41] find a causative relationship between the variabilities of state excise beer taxes and child abuse. Markowitz [38, 39] found a causative link between the price of alcohol on spousal abuse and physical assault by teenagers. Cook and Moore [10] conduct time series analysis of the effects of alcohol prices on crime rates in the USA. Markowitz [36] finds that higher beer taxes are associated with a lower probability of criminal assault. Using state and state alcohol taxes as instruments to deal with the endogeneity issue of alcohol consumption and violence, Corman and Mocan [8] find a causative link between alcohol consumption and assaults. In these studies, the causation runs from the price of alcohol to alcohol consumption, and from alcohol consumption to acts of violence, to violent injury deemed to require medical treatment.

### Other economic factors

Alcohol consumption is by no means the only determinant of violent behaviour.^9^ There exists a vast literature on the link between unemployment and crime. The consensus is of a positive relationship between unemployment and economic crime [31, 50]. However, the relationship between unemployment and violent crime is less certain. The consensus finding has been confirmed for the economic regions of England and Wales in Wu and Wu [63], which shows a strong positive link between unemployment and property crime but an insignificant relationship with violent crime. In contrast, Carmichael and Ward [7] report a positive relationship between youth unemployment and violent crime for the regions of England and Wales. Entorf and Spengler [13] also report a positive relationship between young unemployed persons and assault. Even allowing for the effects of being young and unemployed, simply being young is more strongly associated with certain categories of crime, including rape and assault, which also suggests that a young population be associated with higher violent injury levels.

Income inequality is another important driver of violent crime [62]. Using UN survey data for 45 countries, Fajnzylber et al. [15] find a strong relationship between income inequality and intentional homicide. This finding is also supported for the US by Kelly [27] using granular data at the urban county level and for the England and Wales regions by Wu and Wu [63].

### Environmental factors

Another strand in the literature is the ‘heat hypothesis’ from the field of social psychology. The basic idea is that high temperatures can increase aggression and violent behaviour [1]. Lemon et al. [30] for the UK support this view using the violence surveillance data from the same source as in this paper.^10^ Conversely, it can be argued that wetter weather could dampen the aggressive behaviour accompanying alcoholic consumption. Here we use the average monthly rainfall, and average temperature for each regional economic area to measure potential environmental factors that may affect violent injury independently of all other determinants.

## An analytical framework

Following Markowitz [36], we posit violence to be determined by both the perpetrators’ and victims’ actions. But perpetrators and victims cannot always be distinguished *ex-ante*. In a narrow social setting, such as domestic violence, distinguishing victim from perpetrator is easy. However, in wider social settings (e.g. ‘night clubs’ or ‘sporting venues’), distinguishing them is not always easy. The *ex-post* measure of a victim is the ED entry of a violent injury case (*E*_*i*_) who could be either an ‘innocent’ victim or a perpetrator. The likelihood of being admitted to the ED as a case of violent injury is assumed to be a function of the demand for violence by the individual (which may be zero in the case of an ‘innocent’ victim), the alcohol consumption of the individual admitted to the ED (*A*_*i*_) and that of other individuals in the proximity of the violent action (*A*_*j*_), who could be acquaintances, strangers, or perpetrators, not admitted.

Other driving factors are observed characteristics of admission cases which are correlated with aggregate social, and economic, variables of the region *n* of the respective EDs (*Z*_*n*_) and environmental factors (*X*_*n*_). Substituting for the violence demand function and aggregating, gives an ED violence injury determination equation of the form:

1 $${E}_{nt}=v\left({A}_{nt},{Z}_{nt},{{X}_{n},u}_{nt}\right),$$

where *E*_*nt*_ is the violent injury rate in region *n* at time *t*, *A*_*nt*_ is consumption of alcoholic drinks, *Z*_*nt*_ is a vector of regional social and economic characteristics that correlate with the unobserved individual characteristics of violent injury ED cases, *X*_*n*_ is a vector of local-specific environmental factors, and *u*_*nt*_ is a stochastic component. The violent injury function (10) is augmented by a regional demand for alcoholic drink.

2 $${A}_{nt}=a\left({P}_{Ant},{Y}_{nt},{\Gamma }_{nt},{\varepsilon }_{nt}\right).$$

Here *ε*_*nt*_ is a stochastic term that captures unobserved characteristics,${\Gamma }_{nt}$ is an exogenous variable that drives the demand for alcohol consumption, *Y*_*nt*_ is a regional measure of wealth or income, and *P*_*A*nt_ is the real price of alcohol in the region.^11^ A key exogenous driver is the annual uprating of alcohol duties in April from the Chancellor’s annual Budget Statement.^12^

Equation (1) can be thought of as a violent injury production function. Equation (2) is a regional demand for alcohol. The principal determinants of Eq. (2) are the price of alcohol in the region, country-wide effects (Γ) on alcohol prices, such as changes to alcohol duties and taxes, and other variables associated with alcohol consumption, such as seasonal activity. Substituting Eq. (2) into (1) a reduced form model described by (3) is obtained which shows the direct effect of changes in the price of alcohol on the incidence of violent injury.

3 $${E}_{nt}=f\left({P}_{Ant},{Y}_{nt},{\Omega }_{nt},{\xi }_{nt}\right),$$

where ∂*f*/∂*P*_*Ant*_ < 0, ∂*f*/∂*Y*_*nt*_ > 0, *Ω*_*nt*_ is a vector of other influences {*Γ*_*nt*_, *X*_*nt*_*, Z*_*nt*_} and *ξ*_*nt*_ is a composite error term. Equation (3) states that the price of alcohol has a negative influence on ED violent injury rates. A negative coefficient for alcohol price means that the causation goes from alcohol consumption to violence through to violence-related injury, even if alcohol consumption is an endogenous variable. Nothing suggests that there is two-way causation such that violent injury could cause the price of alcohol. Therefore, the price of alcohol is a strong exogenous driver of violent injury.

## Data

The CSEW is an annual snapshot of crime in general—including micro-information on violent crime. However, seasonal and short-term trend patterns are difficult to infer from these conventional sources. An alternative source is hospital data on people injured by violence (violence-related injury). These data are available at hospital level^13^ and have been collected monthly from computerised records covering January 2005 to December 2014. There are 226 major Emergency Departments (EDs) in England and Wales. Each department collects data (Contract Minimum Data set) on patients. Additionally, computerised departments record whether injuries are accidental or due to interpersonal violence.

**Fig. 1:**
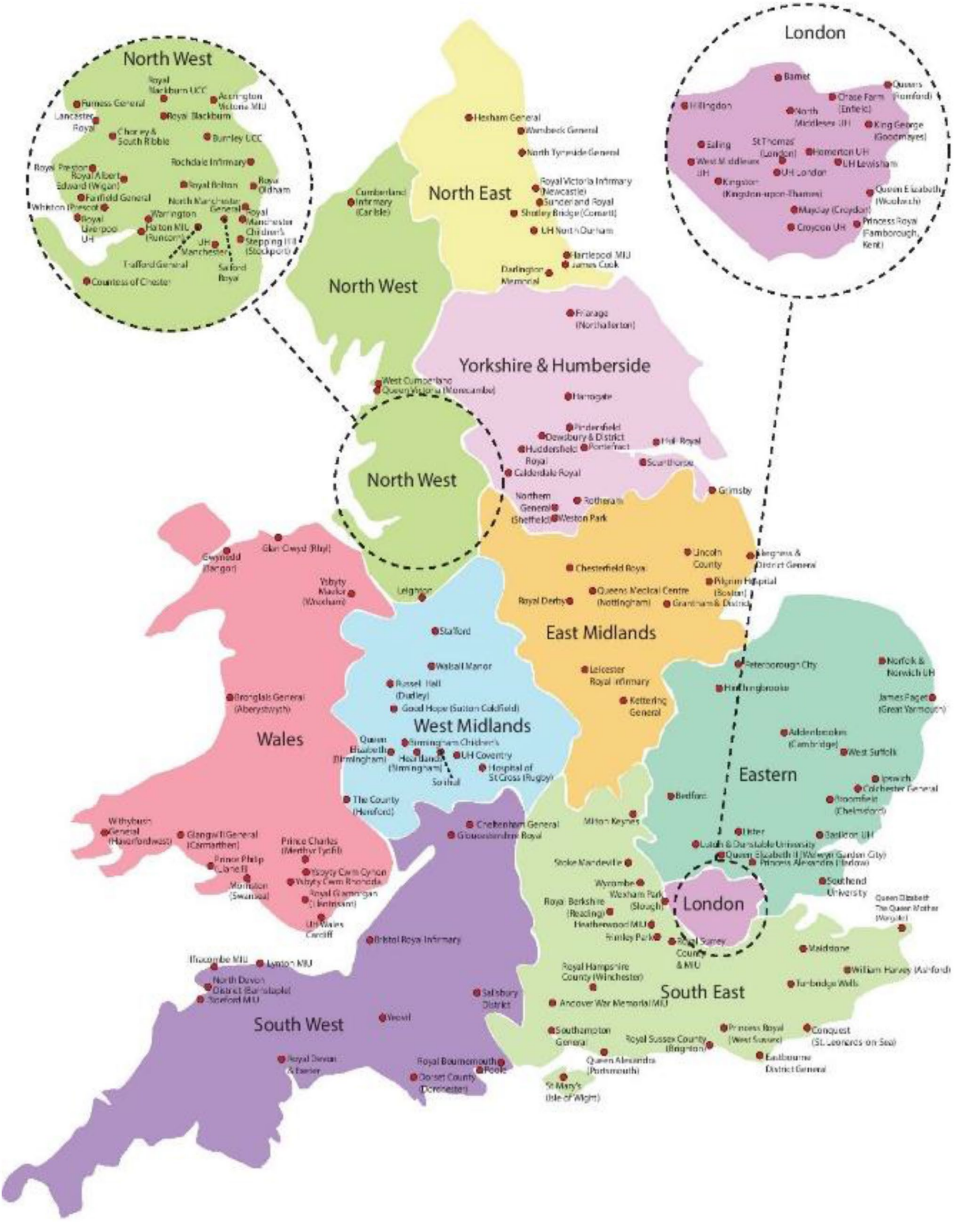
Distribution of EDs (*n* = 166)

The raw anonymised data are obtained from daily records from 166 participating EDs collected from 1 January 2005 to 31 December 2014 and disaggregated by age and gender and were used to derive time series measures of violence-related injury for each *economic* region. Figure 1 shows the geographical distribution of the EDs across England and Wales.

The data are expressed as violent injury admissions per thousand per month for the regional populations. The varying under-representation across regions necessitates a complex weighting strategy. A total number of persons injured by violence were summed across the hospitals within the specific region and weighted by the ratio of the total hospital population in a region to the hospital population of the sample of hospitals in the data frame. Finally, by employing regional resident population figures, the data were expressed as a rate per one thousand of the population.^14^ These data have been used to examine regional trends in monthly violence-related injury and are available by gender and age group [56].^15^ While this weighting strategy provides a clean method of cross-regional comparison, it precludes the comparison of within region hospital violent injury ED rates. In this paper, as a robustness check we go further and examine the data at the hospital level.

**Fig. 2:**
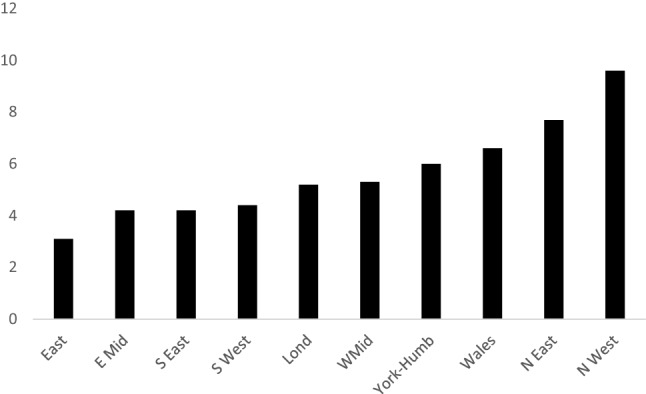
Average Annual Violent Injury rate per 1000, 2005–2014

Figure 2 shows the distribution of the monthly average hospital violent injury rates by region. While these data do not consider under-representation of data reporting in the regions, by expressing the figures as relative to total ED admissions, it provides a revealing geographical snapshot.

The figure shows a clear divide between the relatively depressed post-industrial Northern and Western regions against the low rates in the relatively affluent South and Eastern regions.

On-sale prices for beer, lager, wine, and spirits by economic region were obtained from the Office for National Statistics and used to construct an index of alcohol prices {*P*_*An*_}using expenditure weights obtained from household expenditure on alcohol types from the Department for Environment, Food and Rural Affairs.^16^ The real price of alcohol was obtained by deflating the regional price of alcohol by the national Consumer Price Index (excluding alcohol) and rebased to January 2005 = 100. The same method of construction was followed for the real price of beer, wine, and spirits).^17^

The dependent variable {*E*_*n*_} is the monthly violent injury rate. {*Y*_*n*_} is a regional measure of household wealth/income (proxied by the average real house price). Other independent variables {*Z*_*n*_} include the youth unemployment rate and a measure of regional income inequality. The vector {*X*_n_} comprises measures of local weather and {*Γ*} is a price interaction dummy variable identifying the uprating of the duty on the specific alcoholic drink for each region in April.^18^

Table 1 below describes the data, presents the summary statistics, and provides details of sources. Data for violent injury for 12 months in 2011 for the Northeast and for 2009 West Midlands were not collected. The index of the real price of alcohol and component prices were expressed as relative prices, deflated by the CPI excluding the numerator, and expressed as a natural logarithm. The data on regional house prices were deflated by the CPI.

**Table 1:** Data description and summary statistics Jan 2005–Dec 2014

Variable	Description	N	Mean	Std Dev	Min	Max	Source
VIOLENCE	Monthly Violent Injury rate per one thousand	1176	0.467	0.213	0.076	1.37	VRG Cardiff University
MALE_VIOLENCE	Monthly male Violent Injury rate per one thousand	1176	0.695	0.232	0.129	2.09	VRG Cardiff University
FEMALE_VIOLENCE	Monthly female Violent Injury rate per one thousand	1176	0.247	0.114	0.023	0.794	VRG Cardiff University
MF_VIOLENCE	Monthly male and female Violent Injury rate per one thousand	2352	0.471	0.330	0.023	2.09	VRG Cardiff University
VIOLENCE_ED	Monthly violent injury per one thousand ED attendances	5106	1.260	1.158	0	21.3	VRG Cardiff University
LOG_ALCOHOL PRICE	Logarithm of regional on-sale alcohol prices deflated by the CPI excluding alcohol prices	1200	4.63	0.36	4.57	4.73	Office for National Statistics
LOG_BEER PRICE	Logarithm of regional on-sale beer price deflated by the CPI excluding beer price	1200	4.65	0.035	4.57	4.74	Office for National Statistics
LOG_SPIRIT PRICE	Logarithm of regional on-sale price of spirits deflated by the CPI excluding price of spirits	1200	4.67	0.057	4.56	4.80	Office for National Statistics
LOG_WINE PRICE	Logarithm of regional on-sale price of wines deflated by the CPI excluding price of wines	1200	4.62	0.044	4.52	4.75	Office for National Statistics
LOG_HOUSE PRICE	Logarithm of regional house prices deflated by the CPI	1200	4.50	0.14	4.19	4.86	Nationwide Building Society
YOUTH UNEMP	Regional Youth Unemployment rate	1200	5.35	1.92	1.72	10.6	Office for National Statistics
INEQUALITY	Ratio of regional highest decile earnings to lowest decile	1200	1.49	0.03	1.43	1.56	ASHE: Office for National Statistics
TEMP	Average monthly regional temperature Centigrade	1200	10.45	4.80	− 2.8	22.5	Met Office Hadley Centre Website
RAIN	Average monthly regional rainfall, inches	1200	64.03	40.81	1	286	Met Office Hadley Centre Website

**Fig. 3:**
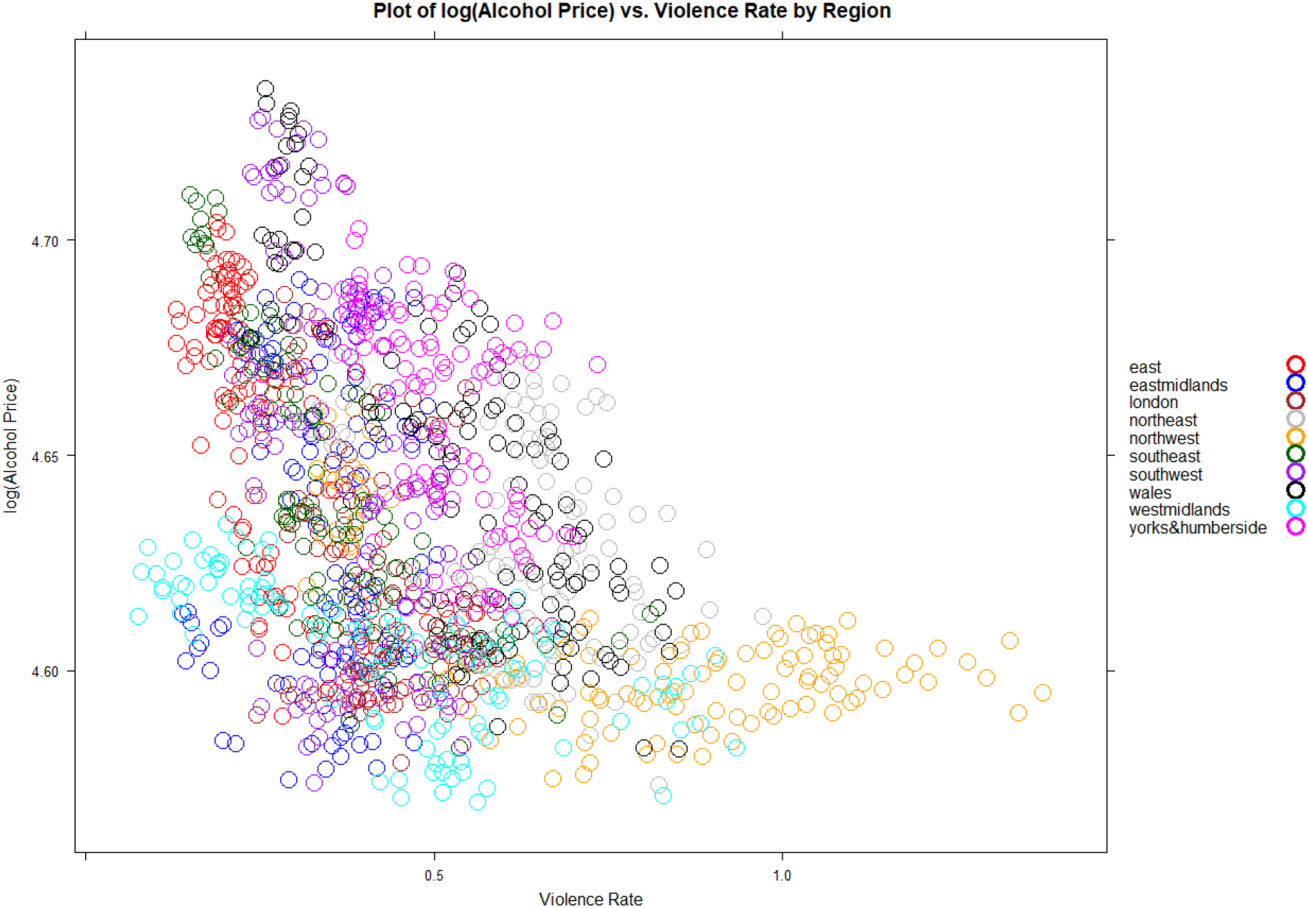
Scatter plot of log of the price alcohol and violence injury rates

**Fig. 4:**
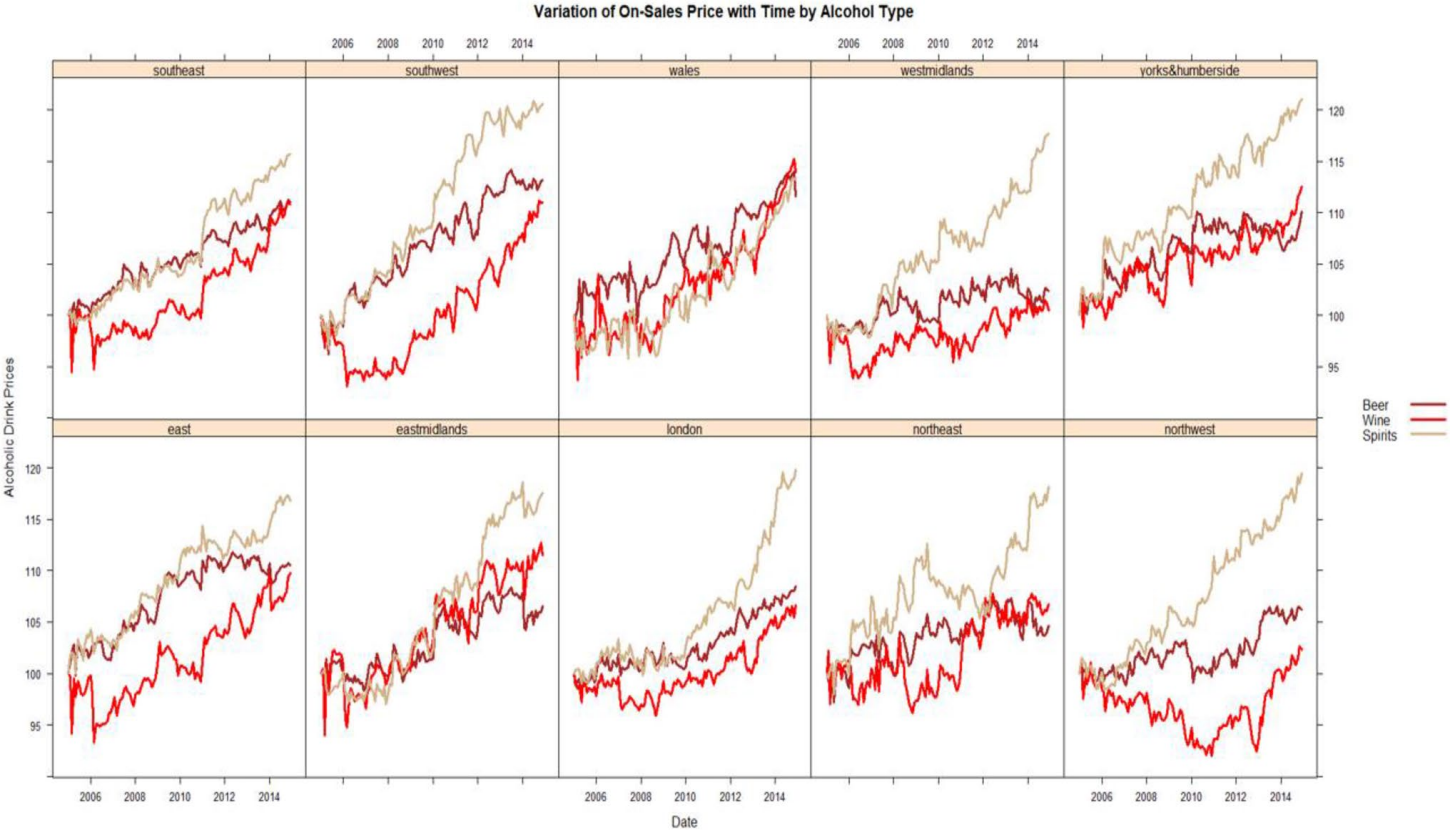
Regional Variation of real price of alcohol by alcohol type 2005.1–2014.12 (2005.1 = 100)

The scatter plot between the log of the real price of alcohol and the regional monthly violence-related injury rates in Fig. 3 reveals a notable visibly inverse relationship with evidently strong heterogeneity in the relationship. For example, a visual inspection shows the north west and west midlands having a strong alcohol price violent injury sensitivity, while Wales shows a price insensitive relation. However, this heterogeneity could be due to observable regional economic and environmental differences as well as unobservable heterogeneities that are modelled using cross-section and time fixed effects in the estimation. Figure 4 shows the time series pattern of the real price of alcohol type for each region. There is clear heterogeneity in the time series variation but in most regions the real price of the three types of alcohol rise over time. But what is striking is the rapid rise in the real price of spirits in all the regions but Wales. We would expect the difference in the movement of the types of alcohol prices to show up in the estimation as we move to differentiate between the effects of these changes.

## Econometric model

The dependent variable is the monthly violent injury rates spanning January 2005–December 2014 for 10 economic regions of England and Wales. The monthly frequency of the data invites dynamic specification as a means of dealing with serial correlation. A second-order lag of the dependent variable was found to work well as an effective dynamic specification. Our first step is to estimate pooled OLS estimates shown in Table 2.

**Table 2:** Pooled OLS; Dependent Variable VIOLENCE; Jan 2005–Dec 2014; mean = .467; Regions = 10; Robust Standard Errors in parenthesis

Variable	1	2	3	4	5	6	7	8	9	10	11	12
VIOLENCE(t-1)	0.590*** (0.028)	0.587*** (0.028)	0.590*** (0.028)	0.589*** (0.028)	0.586*** (0.028)	0.589*** (0.028)	0.591*** (0.028)	0.587*** (0.028)	0.590*** (0.028)	0.592*** (0.028)	0.589*** (0.028)	0.592*** (0.028)
VIOLENCE(t-2)	0.333*** (0.028)	0.337*** (0.028)	0.335*** (0.027)	0.333*** (0.028)	0.336*** (0.028)	0.334*** (0.028)	0.335*** (0.028)	0.338*** (0.028)	0.335*** (0.028)	0.335*** (0.028)	0.338*** (0.028)	0.336*** (0.028)
LOG_ALCHOL PRICE	− 0.133** (0.061)	− 0.100* (0.060)	− 0.089 (0.062)	–	–	–	–	–	–	–	–	–
LOG_BEER PRICE	–	–	–	− 0.139** (0.059)	− 0.110* (0.060)	− 0.098* (0.060)	–	–	–	–	–	–
LOG_SPIRIT PRICE	–	–	–	–	–	–	− 0.077*** (0.039)	− 0.061* (0.039)	− 0.055 (0.039)	–	–	–
LOF_WINE PRICE	–	–	–	–	–	–	–	–	–	− 0.059 (0.049)	− 0.037 (0.050)	− 0.032 (0.059)
LOG_HOUSE PRICE(t-1)	0.056*** (0.021)	0.056*** (0.021)	0.059*** (0.021)	0.059*** (0.021)	0.057*** (0.021)	0.061*** (0.021)	0.056** (0.022)	0.054** (0.022)	0.057*** (0.022)	0.066*** (0.022)	0.063*** (0.022)	0.066*** (0.021)
YOUTH UNEMP	0.004*** (0.001)	0.004*** (0.001)	0.003** (0.001)	0.004*** (0.001)	0.004*** (0.001)	0.003** (0.001)	0.003** (0.001)	0.003** (0.001)	0.003** (0.001)	0.003** (0.001)	0.004** (0.001)	0.003** (0.001)
INEQUALITY	− 0.168** (0.065)	− 0.166** (0.065)	− 0.165** (0.065)	− 0.160** (0.060)	− 0.159** (0.065)	− 0.159** (0.065)	− 0.171*** (0.065)	− 0.167*** (0.065)	− 0.167*** (0.065)	− 0.173*** (0.066)	− 0.168*** (0.066)	− 0.168** (0.065)
TEMP	0.003** (0.001)	0.003** (0.001)	0.002** (0.001)	0.002** (0.001)	0.002** (0.001)	0.002** (0.001)	0.002** (0.001)	0.002** (0.001)	0.002** (0.001)	0.003** (0.001)	0.003** (0.001)	0.002** (0.001)
RAIN	− 0.0001** (0.000)	− 0.0001** (0.000)	− 0.0001** (0.000)	− 0.0001** (0.000)	− 0.0001** (0.000)	− 0.0001** (0.000)	− 0.0001** (0.000)	− 0.0001** (0.000)	− 0.0001** (0.000)	− 0.0001** (0.000)	− 0.0001** (0.000)	− 0.0001** (0.000)
April effect	–	− 0.442*** (0.161)	− 0.502*** (0.171)	–	− 0.439** (0.169)	− 0.499*** (0.170)	–	− 0.517*** (0.160)	− 0.509** (0.186)	–	− 0.478*** (0.161)	− 0.536*** (0.169)
april 2008 effect	–	–	− 0.010** (0.004)	–	–	− 0.010** (0.004)	–	–	− 0.010** (0.004)	–	–	− 0.010** (0.004)
Monthly time dummies	Y	Y	Y	Y	Y	Y	Y	Y	Y	Y	Y	Y
Wald	χ^2^_19_ = 11,068***	χ^2^_20_ = 11,208***	χ^2^_21_ = 11,304***	χ^2^_19_ = 11,094***	χ^2^_20_ = 11,229***	χ^2^_21_ = 11,321***	χ^2^_19_ = 11,037***	χ^2^_20_ = 11,068***	χ^2^_21_ = 11,292***	χ^2^_19_ = 11,028***	χ^2^_20_ = 11,188***	χ^2^_21_ = 11,289***
*N*	1152	1152	1152	1152	1152	1152	1152	1152	1152	1152	1152	1152

***p < 1%

**p < 5%

*p < 10%: Intercept and monthly time dummies not reported

Table 2 shows the base case results using log (real alcohol price) (LOG_ALCOHOL PRICE),^19^ log(real beer price) (LOG_BEER PRICE), log(real spirits price) (LOG_SPIRIT PRICE), and log(real wine price) (LOG_WINE PRICE). The results which include monthly time dummies show a strong negative relationship between the general real price of alcohol, the real price of beer, the real price of spirit, but not wine.

Turning to control variable effects, we can see that the inclusion of real house prices as a proxy for the scale variable in the demand for alcohol is positive suggesting an income effect on the implied demand for alcohol varying by region. Youth unemployment has a positive effect on violent injury—conforming with the findings of Carmichael and Ward [7]. However, income inequality has a negative and statistically significant effect which is not consistent with expectations. That the weather may be a factor in the determination of violent crime has gained credence in the social psychology literature. The inclusion of regional average temperature data from the Met Office shows that this variable is positively related to violent injury. The argument is that warmer weather increases social interaction with the potential for violence independently of alcohol consumption. Similarly, a greater incidence of rainfall is likely to dampen social interaction exerting a negative effect on violent injury. While this latter effect is statistically significant, it is not quantitatively significant as shown in Table 2.

An additional test is to allow for the uprating of duties on alcohol as announced by the Chancellor of the Exchequer in late March and typically brought into force immediately or on 1 April. Often the duty is an uprating for the rate of inflation with some variance on different types of alcohol which is then held fixed until the next budget statement in the following March. However, in Budget 2008, alcohol duties were raised by 6% on 17 March 2008^20^ and have risen 2% in real terms in each year to 2014. While excise duties are ‘expected’ to be uprated for inflation, a 6% rise would be ‘unexpected’ and could have had an independent effect on the demand for alcohol than the ‘expected’ uprating of excise duties.

To test this, we add an interactive term as the product of the time dummy variable for April (unity in April and zero otherwise) and log (real alcohol price and relevant alcohol type). Likewise, we construct an interaction term between an April 2008 dummy variable (unity for April 2008 and zero otherwise) and log (real alcohol price and relevant alcohol type). The results show that there is an ‘April effect’ which supports the notion that exogenous price effects influence the violent injury rate. However, the responsiveness of the violence injury rate to the general real price of alcohol (and both beer and spirits) weakens with the inclusion of the April effect, highlighting the heterogeneous nature of the data and the need to dig deeper into the alcohol price type and gender.

Panel studies of crime typically involve ‘fixed effects’ to account for cross-sectional heterogeneity, and hence, we include fixed effects time dummies to produce two-way fixed effects estimates.^21^ In the second step we report in Table 3 the results from two-way panel fixed effects.

**Table 3:** Panel Estimation 2-Way Fixed Effects; Dependent Variable VIOLENCE; Jan 2005–Dec 2014; mean = 0.467; Regions = 10; Robust Standard Errors in parenthesis

Variable	1	2	3	4	5	6	7	8	9	10	11	12
VIOLENCE(t-1)	0.558*** (0.028)	0.554*** (0.028)	0.557*** (0.028)	0.557*** (0.028)	0.553*** (0.028)	0.556*** (0.028)	0.553*** (0.028)	0.551*** (0.028)	0.553*** (0.028)	0.558*** (0.028)	0.555*** (0.028)	0.557*** (0.028)
VIOLENCE(t-2)	0.313*** (0.028)	0.317*** (0.029)	0.316*** (0.028)	0.312*** (0.028)	0.316*** (0.028)	0.315*** (0.029)	0.310*** (0.028)	0.313*** (0.028)	0.313*** (0.028)	0.314*** (0.028)	0.318*** (0.028)	0.317*** (0.028)
LOG_ALCOHOL PRICE	− 0.132* (0.080)	− 0.089 (0.082)	− 0.076 (0.082)	–	–	–	–	–	–	–	–	–
LOG_BEER PRICE	–	–	–	− 0.175** (0.086)	− 0.142* (0.070)	− 0.121 (0.087)	–	–	–	–	–	–
LOG_SPIRIT PRICE	–	–	–	–	–	–	− 0.148*** (0.055)	− 0.129** (0.056)	− 0.118** (0.056)	–	–	–
LOG_WINE PRICE	–	–	–	–	–	–	–	–	–	− 0.004 (0.067)	0.021 (0.068)	0.024 (0.068)
LOG_HOUSE PRICE(t-1)	0.109*** (0.027)	0.108*** (0.027)	0.112*** (0.027)	0.109*** (0.026)	0.106*** (0.026)	0.145*** (0.027)	0.085*** (0.029)	0.084*** (0.029)	0.089*** (0.047)	0.130*** (0.029)	0.128 (0.029)	
YOUTH UNEMP	0.005*** (0.002)	0.005*** (0.002)	0.005*** (0.002)	0.006*** (0.002)	0.006*** (0.002)	0.008*** (0.002)	0.005*** (0.002)	0.005*** (0.002)	0.004** (0.002)	0.006*** (0.002)	0.006*** (0.002)	
INEQUALITY	0.194 (0.168)	0.194 (0.168)	0.200 (0.167)	0.183 (0.168)	184 (0.167)	0.248 (0.174)	0.164 (0.169)	0.166 (0.168)	0.174 (0.168)	0.210 (0.168)	0.210 (0.168)	
TEMP	0.004*** (0.001)	0.004*** (0.001)	0.004*** (0.001)	0.004*** (0.001)	0.004*** (0.001)	0.004*** (0.001)	0.004*** (0.001)	0.004*** (0.001)	0.004*** (0.002)	0.004*** (0.001)	0.004*** (0.001)	
RAIN	− 0.0001*** (0.000)	− 0.0001*** (0.000)	− 0.0001** (0.000)	− 0.0001*** (0.000)	− 0.0001*** (0.000)	− 0.0001*** (0.000)	− 0.0001*** (0.000)	− 0.0001** (0.000)	− 0.0001** (0.000)	− 0.0001*** (0.000)	− 0.0001*** (0.000)	
April Effect	–	− 0.482*** (0.189)	− 0.539** (0.186)	–	− 0.467** (0.185)	− 0.525*** (0.187)	–	− 0.453** (0.185)	− 0.509** (0.186)	–	− 0.520*** (0.185)	− 0.575*** (0.186)
April 2008 Effect	–	–	− 0.011** (0.004)	–	–	− 0.010** (0.004)	–	–	− 0.010** (0.004)	–	–	− 0.011** (0.004)
*R*^2^ (within)	0.8401	0.8410	0.8419	0.8403	0.8412	0.8420	0.8408	0.8416	0.8424	0.8398	0.8409	0.8418
F for cross-section FE	*F*(9,1123) = 3.6***	*F*(9,1122) = 3.5***	*F*(9,1121) = 3.5***	*F*(9,1123) = 3.8***	*F*(9,1122) = 3.6***	*F*(9,1121) = 3.6***	*F*(9,1123) = 4.4***	*F*(9,1122) = 4.1***	*F*(9,1121) = 3.9***	*F*(9,1123) = 3.7***	*F*(9,1122) = 3.6***	*F*(9,1121) = 3.6***
Hausman χ^2^(r)	31.9(9)	31.2(10)	30.7(11)	33.1(9)	32.0(10)	31.3(11)	38.3(9)	35.7(10)	34.6(11)	32.7(9)	31.8(10)	31.4(11)
N	1152	1152	1152	1152	1152	1152	1152	1152	1152	1152	1152	1152

***p < 1%

**p < 5%

*p < 10%: Intercept and monthly time dummies not reported

A conventional F test of cross-section ‘fixed effects’ against pooled regression with time dummies indicated support for the cross-section fixed effects method.^22^ However, it is well known that including the LDV in a panel model induces biased estimation via its correlation with the error term. In defence, Judson and Owen [27] show this bias to be minor when the time dimension (T) is larger than the cross-section dimension (N). The F test in the last but one row of Table 3 confirms the validity of the panel fixed effects specification. Allowing for cross-sectional heterogeneity weakens the impact of general alcohol prices on violent injury. Except for regional income inequality, the other control variables are significant and have the expected sign. The negative effects from beer and spirits remain along with the April effect, but wine prices have no significant effect.

We have established a negative relationship between the real price of alcohol and violent injury cases and that this runs through all the prices of alcohol except wine. But the statistical significance varies with the type of alcohol price. Is the impact of the significant alcohol prices over-stated? While T is large relative to N, the coefficient on the variable of interest (the real price of alcohol and the price of the specific alcoholic drink) may be upward biased. The results of dynamic estimation to tackle this problem are given in Table 4. Total violent injury rate is regressed, with and without the April effect, on general alcohol price, beer, and spirits. All the regressors saving the lagged dependant variable are assumed exogenous.^23^

**Table 4:** Arellano-Bond Linear Dynamic Panel; Dependent Variable VIOLENCE; Jan 2005–Dec 2014; SE clustered on region id; Regions = 10

Variable	1	2	3	4	5	6
VIOLENCE(t-1)	0.562*** (0.029)	0.560*** (0.021)	0.560*** (0.021)	0.559*** (0.029)	0.557*** (0.021)	0.557*** (0.029)
VIOLENCE(t-2)	0.299*** (0.029)	0.301*** (0.021)	0.298*** (0.021)	0.300*** (0.029)	0.297*** (0.021)	0.299*** (0.021)
LOG_ALCOHOL PRICE	− 0.139* (0.078)	− 0.087 (0.078)	–	–	–	–
LOG_BEER PRICE	–	–	− 0.185** (0.082)	− 0.124 (0.083)	–	–
LOG_SPIRIT PRICE	–	–	–	–	− 0.161*** (0.052)	− 0.125*** (0.053)
LOG_HOUSE PRICE(t-1)	0.137*** (0.027)	0.147*** (0.035)	0.137*** (0.025)	0.141*** (0.025)	0.108*** (0.028)	0.116** (0.037)
YOUTH UNEMP	0.007*** (0.002)	0.008*** (0.002)	0.007*** (0.002)	0.007*** (0.002)	0.006*** (0.002)	0.006*** (0.002)
INEQUALITY	0.078 (0.135)	0.076 (0.135)	0.078 (0.135)	0.077 (0.145)	0.045 (0.135)	0.051 (0.145)
TEMP	0.005*** (0.002)	0.004*** (0.002)	0.005*** (0.001)	0.005*** (0.001)	0.005*** (0.001)	0.005*** (0.001)
RAIN	− 0.0001** (0.000)	− 0.0001** (0.000)	− 0.0001** (0.000)	− 0.0001** (0.000)	− 0.0001** (0.000)	− 0.0001** (0.000)
April effect	–	− 0.489*** (0.168)	–	− 0.479*** (0.168)	–	− 0.456*** (0.168)
April 2008 effect	–	− 0.010*** (0.004)	–	− 0.010*** (0.004)	–	− 0.010*** (0.004)
Time dummies	y	y	y	y	y	y
AR(1–3) test Z Statistic	− 2.9***	− 2.93***	− 2.92***	− 2.93***	− 2.92***	− 2.93***
	− 1.54	− 1.50	− 1.54	− 1.50	− 1.54	− 1.50
	1.78*	1.72*	1.78*	1.72*	1.78*	1.72*
*N*	1152	1152	1152	1152	1152	1152

***p < 1%

**p < 5%

*p < 10%: Intercept and monthly time dummies not reported

The results of Table 4 mirror the findings reported in Table 3 which shows that while a negative relationship is maintained with the general price of alcohol, it is statistically insignificant.^24^ The beer price remains significant but is weakened once the April effect is included. The results must be qualified by the recognition that the ‘April effect’ weakens the overall main price effect for general alcohol prices, but the main results are maintained for beer prices. The ‘April effect’ diminishes in the case of spirits prices but retains a significant 2008 April effect and general April effect. This result underscores the importance of alcohol excise duties on influencing the rate of violent injury. Temperature and rainfall also influence the rate of violent injury.

Tables 2, 3, and 4 show the results based on the total violence injury rates, but part of the heterogeneity in the relationship with alcohol prices could arise from the aggregation of the male and female violence injury rates. The sample mean of the male violence injury rate is nearly three times that of the female violence injury rate. A conventional t test for equality in the sample means is strongly rejected with a value of 44.9. In Table 5, we report the effect of identifying male from female violence injury rates. The data are stacked in a series (and all lagged variables generated and inputted separately) effectively doubling the data points. Regional and time dummies were included to capture cross-section and time varying heterogeneity. A gender interaction term is generated (GENDER = 1 for male violence injury and 0 for female violence injury) with the log of the specific alcohol price included in the regression model (LOG_ALCOHOL PRICE*GENDER, or the log of the price of the alcohol type*GENDER). This is shown in Table 5 as the GENDER EFFECT. Allowing for this gender heterogeneity, we find stronger effects of alcohol prices on violent injury. As expected, the gender dummy is positive and strongly significant, and the gender effect shows that the responsiveness of male violence injury to alcohol prices (and type of alcohol price) dominates that of female. The April effect remains strong.

**Table 5:** Panel Estimation 2-Way Fixed Effects; Dep Var MF_VIOLENCE; Jan 2005–Dec 2014; mean = 0.471, Regions 10; Robust Standard Errors in parenthesis

Variable	1	2	3	4	5	6	7	8
MF_VIOLENCE(t-1)	0.558*** (0.030)	0.558*** (0.030)	0.558*** (0.030)	0.558*** (0.030)	0.557*** (0.030)	0.556*** (0.030)	0.561*** (0.030)	0.560*** (0.030)
MF_VIOLENCE(t-2)	0.325*** (0.031)	0.326*** (0.031)	0.324*** (0.031)	0.326*** (0.031)	0.324*** (0.031)	0.325*** (0.031)	0.328*** (0.031)	0.329*** (0.031)
LOG_ALCOHOL PRICE	0.040 (0.069)	0.083 (0.069)	–	–	–	–	–	–
LOG_BEER PRICE	–	–	− 0.000 (0.072)	0.051 (0.074)	–	–	–	–
LOG_SPIRIT PRICE	–	–	–	–	− 0.042 (0.046)	− 0.014 (0.046)	–	–
LOG_WINE PRICE	–	–	–	–	–	–	0.091 (0.060)	0.119** (0.060)
GENDER MALE = 1	1.73*** (0.410)	1.71*** (0.409)	1.77*** (0.429)	1.75*** (0.428)	1.00*** (0.271)	1.00*** (0.271)	1.17*** (0.339)	1.16*** (0.337)
GENDER Effect	− 0.362*** (0.088)	− 0.359*** (0.087)	− 0.371*** (0.092)	− 0.367*** (0.091)	− 0.224*** (0.057)	− 0.224*** (0.057)	− 0.243*** (0.073)	− 0.242*** (0.072)
LOG_HOUSE PRICE(t-1)	0.092*** (0.025)	0.094*** (0.025)	0.092*** (0.024)	0.093*** (0.023)	0.064** (0.027)	0.064** (0.027)	0.106** (0.027)	0.107** (0.026)
YOUTH UNEMP	0.004*** (0.002)	0.004*** (0.002)	0.005*** (0.002)	0.005*** (0.002)	0.004*** (0.002)	0.004*** (0.002)	0.004*** (0.002)	0.004*** (0.002)
INEQUALITY	0.185 (0.154)	0.193 (0.152)	0.172 (0.154)	0.182 (0.153)	0.147 (0.153)	0.147 (0.153)	0.197 (0.153)	0.202 (0.153)
TEMP	0.004*** (0.001)	0.004*** (0.001)	0.004*** (0.001)	0.004*** (0.001)	0.004*** (0.001)	0.004*** (0.001)	0.004*** (0.001)	0.004*** (0.001)
RAIN	− 0.0001*** (0.000)	− 0.0001*** (0.000)	− 0.0001*** (0.000)	− 0.0001*** (0.000)	− 0.0001*** (0.000)	− 0.0001*** (0.000)	− 0.0001*** (0.000)	− 0.0001*** (0.000)
April effect	–	− 0.548*** (0.139)	–	− 0.538*** (0.139)	–	− 0.522*** (0.139)	–	− 0.583*** (0.138)
April 2008 effect	–	− 0.011*** (0.003)	–	− 0.010*** (0.003)	–	− 0.010*** (0.003)	–	− 0.011*** (0.003)
*R*^2^	0.9457	0.9461	0.9458	0.9461	0.9458	0.9462	0.9455	0.9460
F test for cross-section FE	*F*(9,2276) = 4.66***	*F*(9,2274) = 4.69***	*F*(9,2276) = 5.13***	*F*(9,2274) = 4.69***	*F*(9,2276) = 5.60***	*F*(9,2274) = 5.17***	*F*(9,2276) = 4.64***	*F*(9,2274) = 4.21***
*N*	2304	2304	2304	2304	2304	2304	2304	2304

***p < 1%

**p < 5%

*p < 10%; Intercept not shown

In summary, alcohol prices exert a negative effect on the violent injury rate, and its statistical significance depends on alcohol type. The real price of wine does not appear to play any part in determining violent injury rate but the price of spirits and to a lesser extent the price of beer do. There is a significant gender effect with alcohol prices affecting male violent injury rates more than those of female. There is a significant April effect and an additional April 2008 effect. Weather conditions play a part as do local economic conditions measured by youth unemployment and local house prices.

## Robustness tests

Here we report robustness tests by using the data from individual hospitals which comprise raw admissions recorded as violent injury divided by the total of admissions for 166 Emergency Departments in England and Wales. Reporting inconsistencies mean that hospitals did not always provide continuous data. In other cases, hospitals were closed or merged with others to create a new entity. Emergency Departments with only 12 months of data were removed from the sample. The sample is unbalanced and consisted of 5094 usable monthly ED observations. A balanced panel of 10 ED hospitals for 36 months could be extracted from the data but the sample would be too small for valid statistical inference.

First, we use raw hospital data matched to economic region. Second, we examine the gender effect by separating male and female violent injury rates. Table 6 presents the results of panel two-way fixed effects estimates using the hospital as the cross-sectional dimension and with standard errors clustered around hospital ID. Because of the unbalanced nature of the raw data the April 2008 effect was excluded as there were only 47 observations.^25^ The first column shows results for the general price of alcohol without the April effects and the second column shows the inclusion of the April effect. The results are consistent with the main results with respect to the different prices of alcohol. The youth unemployment rate and inequality have lost statistical significance, but weather factors maintain significance as expected.

**Table 6:** Panel 2-Way Fixed Effects; Dep Var = VIOLED monthly violent injury rate per ED; Jan 2005–Dec 2014, EDs = 166; SE clustered on hospital id

Variable	1	2	4		5	4	5	4	5
VIOLED(t-1)	0.478*** (0.014)	0.478*** (0.013)	0.479*** (0.014)		0.478*** (0.014)	0.476*** (0.015)	0.475*** (0.015)	0.479*** (0.013)	0.479*** (0.014)
VIOLED(t-2)	0.267*** (0.032)	0.267*** (0.032)	0.267*** (0.032)		0.267*** (0.032)	0.265*** (0.031)	0.266*** (0.031)	0.267*** (0.033)	0.268*** (0.132)
LOG_ALCOHOL PRICE	− 0.693** (0.351)	− 0.622* (0.346)	–		–	–	–	–	–
LOG_BEER PRICE	–	–	− 0.702* (0.371)		− 0.647* (0.368)	–	–	–	–
LOG_SPIRIT PRICE	–	–	–		–	− 0.933*** (0.236)	− 0.872*** (0.238)	–	–
LOG_WINE PRICE	–	–	–		–	–	–	− 0.231 (0.306)	− 0.167 (0.277)
April Effect	–	− 10.324*** (0.346)	–		− 0.889 (0.614)	–	− 754** (0.369)	–	− 10.12** (0.453)
LOG_HOUSE PRICE(t-1)	0.501*** (0.146)	0.496*** (0.145)	0.526*** (0.154)		0.525*** (0.107)	0.312** (0.157)	0.312** (0.1237)	0.566*** (0.138)	0.560*** (0.112)
YOUTH UNEMP	0.006 (0.006)	0.006 (0.007)	0.008 (0.006)		0.008 (0.007)	− 0.001 (0.007)	0.0001 (0.008)	0.006 (0.007)	0.006 (0.008)
INEQUALITY	10.37* (0.758)	10.38* (0.756)	10.34* (0.753)		10.34** (0.677)	0.960 (0.766)	0.968 (0.685)	0.960 (0.766)	10.46** (0.674)
TEMP	0.018*** (0.003)	0.018*** (0.003)	0.018*** (0.003)		0.017*** (0.004)	0.018*** (0.003)	0.018*** (0.004)	0.018*** (0.003)	0.018*** (0.004)
RAIN	− 0.0006*** (0.000)	− 0.0005*** (0.000)	− 0.0006*** (0.000)		− 0.0005*** (0.000)	− 0.0006*** (0.000)	− 0.0006*** (0.000)	− 0.0006*** (0.000)	− 0.0005*** (0.000)
*R*^2^(within)	0.5717	0.5722	0.5717		0.5721	0.5728	0.5732	0.5713	0.5719
Test for cross-section FE	*F*(164,4423) = 1.60***	*F*(164,4422) = 1.59***	*F*(164,4423) = 1.61***		*F*(164.4422) = 1.60***	*F*(164,4423) = 1.63***	*F*(164,4422) = 1.62***	*F*(164,4423) = 1.63***	*F*(164,4422) = 1.62***
Hausman *χ*^2^(*r*)	251.0(14)	247.9(16)	252.1(14)		250.7(16)	255.4(14)	254.2(16)	256.2(14)	254.1(16)
*N*	4607	4607	4607		4607	4607	4607	4607	4607

As a third test for robustness, in Table 7 we report the results from disaggregating the violent injury data at the ED level by gender with respect to the different types of alcohol prices including the April effects.^26^ The results confirm that the strongest effect is on the male violence injury rates. In the case of female injury rates, only the price of spirits has a significant effect. The April effect is mostly retained.^27^

**Table 7:** Panel Estimation 2-Way Fixed Effects; Dependent Variable = VIOLED Monthly Violent Injury per ED; Jan 2005–Dec 2014; EDs = 166; SE clustered on hospital id

Variable	Male violence injury rates; mean = 0.9025				Female violence injury rates; mean = 0.3333			
	1	2	3	4	5	6	7	8
VIOLED(t-1)	0.424*** (0.017)	0.424*** (0.017)	0.419*** (0.016)	0.425*** (0.017)	0.494*** (0.075)	0.494*** (0.075)	0.492*** (0.077)	0.494*** (0.075)
VIOLED(t-2)	0.273*** (0.023)	0.273*** (0.014)	0.271*** (0.021)	0.274*** (0.023)	0.132*** (0.077)	0.132*** (0.078)	0.130*** (0.076)	0.133*** (0.078)
LOG_ALCOHOL PRICE	− 0.688** (0.326)	–	–	–	− 0.152 (0.147)	–	–	–
LOG_BEER PRICE	–	− 0.652** (0.311)	–	–	–	− 0.141 (0.189)	–	–
LOG_SPIRIT PRICE	–	–	− 0.842*** (0.207)	–	–	–	− 0.283** (0.038)	–
LOG_WINE PRICE	–	–	–	− 0.227 (0.279)	–	–	–	− 0.021 (0.119)
LOG_HOUSE PRICE(t-1)	0.463*** (0.126)	0.486*** (0.130)	0.297** (0.130)	0.519*** 0.121)	0.156*** (0.049)	0.163*** (0.050)	0.087 (0.062)	0.177*** (0.044)
YOUTH UNRMP	0.007 (0.006)	0.010 (0.005)	0.002 (0.006)	0.008 (0.006)	− 0.0001 (0.003)	− 0.0002 (0.003)	− 0.003 (0.003)	− 0.000 (0.003)
INEQUALITY	10.21* (0.646)	10.19* (0.645)	0.847 (0.657)	10.29** (0.645)	0.567** (0.272)	0.563** (0.265)	0.428* (0.259)	0.589 (0.278)
TEMP	0.013*** (0.002)	0.012*** (0.002)	0.013*** (0.002)	0.013*** (0.002)	0.005*** (0.002)	0.005*** (0.002)	0.005*** (0.002)	0.005*** (0.002)
RAIN	− 0.0005*** (0.000)	− 0.0004** (0.000)	− 0.0004** (0.000)	− 0.0004** (0.000)	− 0.0001* (0.000)	− 0.0001* (0.000)	− 0.0001* (0.000)	− 0.0001* (0.000)
April Effect	− 0.759*** (0.251)	− 0.662*** (0.261)	− 0.680*** (0.262)	− 0.816*** (0.252)	− 0.509* (0.287)	− 0.514* (0.296)	− 0.475 (0.296)	− 0.528* (0.278)
*R*^2^ (Within)	0.5275	0.5274	0.5291	0.5270	0.3934	0.3934	0.3945	0.3933
*F* test for cross-section fixed effects	*F*(163,4398) = 1.98***	*F*(163,4398) = 1.99***	*F*(163,4398) = 2.01***	*F*(163,4398) = 2.01***	*F*(161,4358) = 1.89***	*F*(161,4358) = 1.88***	*F*(161,4358) = 1.88***	*F*(161,4358) = 1.93***
*N*	4582	4582	4582	4582	4540	4540	4540	4540

***p < 1%

**p < 5%

*p < 10%; intercept not shown

## Discussion

We present an econometric model of the determination of violence-related injuries incorporating economic and environmental factors. What do we know about the determinants of violent injury from this empirical exercise? We can separate the determinants into environmental factors and economic factors. In terms of geographical environmental factors, the weather makes a small but significant contribution to the regional heterogeneity in violent injury rates. Economic factors can be separated into short-term policy instruments and longer-term regional economy influences. First, it can be concluded that the rate of violence-related injury is inversely related to the real price of alcohol, and this is particularly strong for spirits prices, but not wine. Second, we find a strong April effect coinciding with the annual uprating of excise duties on alcohol prices. Third, the relationship between alcohol prices and violent injury is particularly strong for male violent injury. Fourth, regional economic conditions play a significant part in the determination of violent injury although inequality has a weaker and ambiguous effect.

While these ‘big picture’ factors of regional economic differences are strong drivers of violent injury, our main conclusion is that raising the real price of alcoholic drink and particularly that of spirits would have a significant downward effect on the violence-related injury rate. Specifically, this paper highlights the role for taxation as a second-best solution to the negative welfare effects of violent injury admissions to EDs in the UK health service. A large literature establishes the link between alcohol prices and taxes on alcoholic drinking and drunkenness. It is argued that tax effects are large compared with other forms of prevention policies and programmes [60]. Increased taxes on alcohol will not only reduce alcohol consumption and reduce the costs associated with a pressing social and health problem but also have the positive externality of reducing violence and violence-related injury.

It is arguable that the first-best policy relating to a reduction in violent injury admissions is the increase in the price of violence. However, there are costs associated with violent injury reduction through stronger policing and increased penalties. The policy implications of such a move are not discussed in this paper, but it is recognised that such an action may involve a protracted political process. It is also the case that the government recognises the trade-off between resources allocated to violent injury reduction and other governmental activity. An easier and politically more tractable action is to levy taxes which may now be possible given independence from any future European Directives.

So, what does an increase in alcohol taxes that raises the average real price of alcohol by 1% imply? The average ED violence injury rate for the full sample is 0.467 per one thousand of the population of England and Wales a month. From the parameter estimates of column 1 of Tables 3, 4, we obtain the semi-elasticity of violent injury with respect to general alcohol prices in the range − 1.0 to − 1.35. Taking the lower of these figures as a conservative estimate, we can calculate the long-run effect of a 1% rise in the real price of alcohol on the violence injury rate per month to reduce the average violence injury rate by approximately 0.01 per thousand per month. In 2014, the total population of England and Wales was 58.4 million.^28^ A sustained 1% rise in real alcohol prices reduces violent injury attendances at EDs by approximately 7000 a year.^29^

Heeks et al. [24] calculate the unit cost of violence with injury at £14,050 (Table E1, p7). This cost is the sum of the anticipation of crime (costs incurred by the victim to avoid violent injury), consequence of crime (cost of physical damage to the person), and response to crime (costs to the police and criminal justice system). Applying these costs to the marginal reduction in ED admissions from a sustained 1% rise in the real price of alcohol gives a cost saving of nearly £98 million a year. This figure does not consider the cost saving to the NHS from reduced ED attendances or the revenue gains to the exchequer from the increase in duties.

How might these results change with the two big economic events since the end of the data sample—Brexit and the COVID-19 pandemic? The Brexit effect on the trade of alcoholic drinks from the EU would have come into effect at about the same time as the pandemic restrictions on economic activity and would therefore be hard to disentangle. However, as the effect on imported wines from the EU would be not subjected to any additional tariff, the change in the price would reflect the depreciation of the exchange rate. Our results show that violent injury rates are not responsive to real wine price changes, and we surmise that the Brexit effect would be minimal. However, the pandemic resulted in a 23% drop in violent injury reported to hospitals between April and July 2020.^30^ Although there was no discernible differential gender effect in the aggregate data further work on an extended data set could explore the potential of hidden domestic violence in the gender violent injury statistics.

## Supplementary Information

Below is the link to the electronic supplementary material.

Supplementary file1 (DOCX 24 KB)

## Data Availability

The violence injury data are collected from individual EDs and collated at the Violence Research Group, Cardiff University (https://www.cardiff.ac.uk/violence-research-group). Data and estimation codes are available on request from the corresponding author.

## Footnotes

1 See Pernanen [47, 48].

2 Similarly, Anderson et al. [2] finds evidence that a 10% increase in alcohol consumption outlets in counties of Kansas that moved from ‘dry’ to ‘wet’ increased violence 3–5%,

3 Lipsey et al. [33].

4 Markowitz [37] also examines the relationship between international violence rates in terms of the differences in the price of alcohol.

5 Matthews et al. [42] examine the effect of the price of beer on violent injury, and Page et al. [46] examine the effect of on-licenced and off-licenced alcohol prices. Both models are static and do not allow for dynamic effects.

6 The 1996 British Crime Survey included a computerised self-completion questionnaire designed to guarantee anonymity and measure the extent of domestic violence [45].

7 For recent surveys see Boden et al. [4], Fitterer et al. [18], Leonard and Quigley [29], Brown and Leonard [5], and Garofalo and Wright [19].

8 For example, high alcohol consumption and violent behaviour have been associated with young people exposed to social disadvantage, dysfunctional families, and parental deviance.

9 Stronger effects from measures of poverty and income inequality are reported in Page et al. [47] in addition to alcohol prices.

10 Other supporting evidence for the US is reported in Michael et al. [44].

11 Recent evidence on the own price effects on alcohol consumption in the UK includes Sousa [58], Meng et al. [43], and Connolly et al. [9].

12 Evidence for the US shows that alcohol taxation reduces alcohol demand and alcohol related harm. See Elder et al. [11].

13 ED data have been highlighted as an alternative source of violent injury by Sivarajasingham et al. [57] and applied recently in Liasidou and Gregoriou [28].

14 As an example, the January 2005 raw data of violent injury admissions across all hospitals of the East region were 261. Weighting by the hospital coverage ratio gives a figure of 2329. The regional population was 5,563,200. The monthly rate per one thousand of the regional population is constructed as (2329/5563200)*1000 = 0.4.

15 Additional details of the construction of regional data can be found in Sivarajasingham et al. [57].

16 http://webarchive.nationalarchives.gov.uk/20130103014432/http://www.defra.gov.uk/statistics/foodfarm/food/familyfood/datasets/.

17 The choice of the CPI (excluding alcohol) as the deflator was dictated by the expenditure weights used to construct the regional alcohol price consistent with the ONS weights. The parameter estimates are sensitive to an alternative deflator such as the RPI (excluding alcohol) but qualitatively produce the same results as in Table 2 and 3. One implication of working with real prices is that changes to VAT do not materially affect the real price which is a relative price. However, there were two incidences of alcohol duties being changed outside the months March–April within this sample period. They were December 2008 and October 2011. We investigated the possible shocks to the real price of beer, spirit, and wine, and in all cases the effects were not statistically significant. We thank an anonymous referee for alerting us to this.

18 An ‘April only’ dummy variable would not work because the model includes monthly time dummies. A proxy for the ‘April effect’ is the interaction term of the April dummy variable and the on-sales price of alcohol, or the specific alcoholic drink (beer, spirits, wine).

19 Confirming the findings of Page et al. [46].

20 HM Treasury [25]-

21 See for example Entorf and Spengler [13] and Machin and Meghir [35].

22 Following Gosh and Gregoriou [22] we also report the Hausman test for the determination of the FE estimator. In all cases the FE estimator was accepted.

23 The results for wine were consistent with Tables 2 and 3 and not reported.

24 The estimated parameters on the price of alcohol in Table 4 are not far different from the estimates of Table 3, confirming the finding of Judson and Owen [26].

25 The April 2008 was also statistically insignificant.

26 In 44 cells of the hospital data sample there were no female violence injuries reported, and in 20 cells there were no male violence injuries reported.

27 As a final robustness test, we report selected results of Seemingly Unrelated Regression estimation of the pooled and two-way fixed effects estimation results of Tables 2 and 3 as in Ashton et al. [3] and selected two-step GMM estimation of Table 4 as requested by an anonymous reviewer. The results largely support those reported in the main body.

28 https://www.ons.gov.uk/peoplepopulationandcommunity/populationandmigration/populationestimates/bulletins/annualmidyearpopulationestimates/2015-06-25

29 Figure 2 indicates the geographical distribution of this estimate over England and Wales.

30 https://www.cardiff.ac.uk/__data/assets/pdf_file/0003/2620335/Violence-in-England-and-Wales-in-2021-NVSN-Report-2022.pdf
