# Supplementary material for: Alcohol prices, the April effect, and the environment, in violence-related injury in England and Wales
Source: Eur J Health Econ. 2023 Mar 29;25(2):237–55. doi: 10.1007/s10198-023-01583-w (PMC10052256; doi:10.1007/s10198-023-01583-w)
Supplement: Supplementary file 1 — Supplementary file1 (DOCX 24 KB) [file 10198_2023_1583_MOESM1_ESM.docx]

**Appendix**

This appendix to the main paper presents further robustness test at the request of two anonymous referees. In the first robustness test we re-estimate the equations of Tables 2 and 3 with Seemingly Unrelated Regression with the objective of obtaining greater precision of the standard errors. Table 1A shows selected results of the pooled regression with time fixed effects and the 2-way fixed effects. Columns 1-3 show the results from the models of columns 1-3 of Table 2. There is no discernible improvement in the precision of the estimates. Columns 4-7 show the results of the models of columns 3, 6, 9, and 12 of Table 3. Again, there is no discernible improvement in the results.

**Table 1A: Seemingly Unrelated Regression Panel Estimation; Dependent Variable VIOLENCE; jan-2005 – dec-2014; mean = .467; Regions = 10**

| Variable | 1 | 2 | 3 | 4 | 5 | 6 | 7 |
| --- | --- | --- | --- | --- | --- | --- | --- |
| VIOLENCE(t-1) | .589***  (.028) | .585***  (.028) | .587***  (.028) | .557***  (.028) | .555***  (.028) | .553***  (.028) | .5579***  (.028) |
| VIOLENCE(t-2) | .346***  (.027) | .350***  (.027) | .348***  (.027) | .316***  (.028) | .314***  (.029) | .311***  (.028) | .316***  (.028) |
| LOG_ALCOHOL PRICE | -.142**  (.065) | -.103  (.066) | -.092  (.066) | -.076  (.081) | - | - | - |
| LOG_BEER PRICE | - | - | - | - | -.123  (.086) | - | - |
| LOG_SPIRIT PRICE | - | - | - | - | - | -.120**  (.056) | - |
| LOG_WINE PRICE | - | - | - | - | - | - | .026  (.067) |
| LOG_HOUSE PRICE(t-1) | .055***  (.022) | .055***  (.022) | .059***  (.021) | .112***  (.027) | .112***  (.025) | .090***  (.029) | .130***  (.028) |
| YOUTH UNEMP | .003***  (.001) | .003***  (.001) | .003**  (.001) | .005***  (.002) | .006***  (.002) | .004**  (.002) | .005***  (.002) |
| INEQUALITY | -.152**  (.072) | -.150**  (.071) | -.152**  (.071) | .200  (.166) | .188  (.166) | .171  (.166) | .215  (.165) |
| TEMP | .003**  (.001) | .003**  (.001) | .002*  (.001) | .004***  (.001) | .004***  (.001) | .004***  (.001) | .004***  (.001) |
| RAIN | -.0001**  (.000) | -.0001*  (.000) | -.0001*  (.000) | -.0001**  (.000) | -.0001***  (.000) | -.0001**  (.000) | -.0001**  (.000) |
| April Effect | - | -.517***  (.185) | -.577***  (.186) | -.539***  (.184) | -.482***  (.185) | -.239**  (.111) | -.575***  (.186) |
| April 2008 Effect | - | - | -.052**  (.021) | -.05**  (.02) | -.009**  (.004) | -.010**  (.004) | -.011**  (.004) |
| R^2^ | .91776 | .9182 | .9186 | .9208 | .9208 | .9209 | .9208 |
| Panel 2-way FE | N | N | N | Y | Y | Y | Y |
| N | 1152 | 1152 | 1152 | 1152 | 1152 | 1152 | 1152 |

*** p < 1%; ** p < 5%; * p < 10%: Intercept not reported.

In Table 2A we present the results from system GMM for the weighted real price of alcohol and the real price of spirits. The results are qualitatively like the findings of Table 4 in the text.

**Table 2A: GMM One-Step System Estimation 2-Way Fixed Effects; Dependent Variable VIOLENCE; jan-2005 – dec-2014; mean = .467; Regions = 10;**

| Variable | 1 | 2 | 3 | 4 |
| --- | --- | --- | --- | --- |
| VIOLENCE(t-1) | .549***  (.028) | .548***  (.027) | .546***  (.028) | .546***  (.027) |
| VIOLENCE(t-2) | .306***  (.028) | .308***  (.030) | .303***  (.030) | .304***  (.029) |
| LOG_ALCOHOL PRICE | -.144**  (.066) | -.099  (.139) | - | - |
| LOG_SPIRIT PRICE | - | - | -.145**  (.071) | -.117*  (.078) |
| LOG_HOUSE PRICE(t-1) | .109**  (.045) | .058***  (.022) | .086**  (.034) | .092**  (.037) |
| YOUTH UNEMP | .006***  (.002) | .003**  (.001) | .005***  (.002) | .003**  (.001) |
| INEQUALITY | .317**  (.144) | -.152**  (.072) | .292*  (.151) | .005***  (.002) |
| TEMP | .004***  (.001) | .002*  (.001) | .004***  (.001) | .004***  (.001) |
| RAIN | -.0001**  (.000) | -.0001**  (.000) | -.0001***  (.000) | -.0001***  (.000) |
| April Effect | - | -.519***  (.175) | - | -.237**  (.115) |
| April 2008 Effect | - | -.050***  (.012) |  | -.010***  (.002) |
| AR(1) in differences z | -2.89*** | -2.90*** | -2.89*** | -2.91*** |
| AR(2) in differences z | -1.71* | -1.67* | -1.72* | -1.65* |
| Sargan p(H0:v) | 0.27 | 0.26 | 0.26 | 0.26 |
| N | 1140 | 1140 | 1140 | 1140 |

*** p < 1%; ** p < 5%; * p < 10%: Intercept and monthly time dummies not reported
